# The epidemiology of fighting in group-housed laboratory mice

**DOI:** 10.1038/s41598-020-73620-0

**Published:** 2020-10-06

**Authors:** Jacob H. Theil, Jamie Ahloy-Dallaire, Elin M. Weber, Brianna N. Gaskill, Kathleen R. Pritchett-Corning, Stephen A. Felt, Joseph P. Garner

**Affiliations:** 1 Campus Veterinary Services, University of California, Davis, One Shields Ave., Davis, CA 95616 USA; 2 Département des Sciences Animales, Université Laval, Quebec, QC G1V 0A6 Canada; 3 Department of Animal Environment and Health, Swedish University of Agricultural Sciences, Gråbrödragatan 19, 532 31 Skara, Sweden; 4 Animal Sciences Department, Purdue University, 270 S. Russell St., West Lafayette, IN 47907 USA; 5 Office of Animal Resources, Harvard University Faculty of Arts and Sciences, 16 Divinity Ave., Cambridge, MA 02138 USA; 6 Department of Comparative Medicine, Stanford University, 300 Pasteur Drive, Stanford, CA 94305-5342 USA; 7 (By Courtesy), Department of Psychiatry and Behavioral Sciences, Stanford University, 300 Pasteur Drive, Stanford, CA 94305-5342 USA

**Keywords:** Animal behaviour, Animal physiology

## Abstract

Injurious home-cage aggression (fighting) in mice affects both animal welfare and scientific validity. It is arguably the most common potentially preventable morbidity in mouse facilities. Existing literature on mouse aggression almost exclusively examines territorial aggression induced by introducing a stimulus mouse into the home-cage of a singly housed mouse (i.e. the resident/intruder test). However, fighting occurring in mice living together in long-term groups under standard laboratory housing conditions has barely been studied. We performed a point-prevalence epidemiological survey of fighting at a research institution with an approximate 60,000 cage census. A subset of cages was sampled over the course of a year and factors potentially influencing home-cage fighting were recorded. Fighting was almost exclusively seen in group-housed male mice. Approximately 14% of group-housed male cages were observed with fighting animals in brief behavioral observations, but only 14% of those cages with fighting had skin injuries observable from cage-side. Thus simple cage-side checks may be missing the majority of fighting mice. Housing system (the combination of cage ventilation and bedding type), genetic background, time of year, cage location on the rack, and rack orientation in the room were significant risk factors predicting fighting. Of these predictors, only bedding type is easily manipulated to mitigate fighting. Cage ventilation and rack orientation often cannot be changed in modern vivaria, as they are baked in by cookie-cutter architectural approaches to facility design. This study emphasizes the need to invest in assessing the welfare costs of new housing and husbandry systems before implementing them.

## Introduction

Mouse welfare has advanced in leaps and bounds in recent years—a biologically relevant^1^ and easily implementable enrichment (nesting material^2–10^) is now widely adopted; and a 95% effective cure for ulcerative dermatitis (which is the most common morbidity in mice, and was previously intractable^11^) has been identified. This now leaves injurious home-cage aggression (fighting) as the primary welfare issue in mice in terms of potentially preventable morbidity, comprising roughly 15% of morbidities observed in mice in one prevalence study^12^, with male mice bearing the brunt of this burden^13–16^.

Of necessity, mice used in research are housed in cages, and current caging typically prevents normal submissive behavioral responses to aggression, such as flight or retreat from sight. This disruption to normal behavior increases the likelihood that agonistic encounters will escalate into fighting, and potential injury. With or without injury, the stress of such interactions impacts welfare, alters mouse physiology, and introduces significant experimental confounds^15,16^. When mice are injured, welfare and scientific concerns are even more severe^15–18^. Severe trauma may require early euthanasia of individuals, reducing the power of the studies to which they are assigned. Additionally, managing fighting animals requires medical intervention and separation, which can be expensive for investigators and facilities, and increases study variability. Altogether, conspecific aggression is a significant welfare concern and scientific confound, and identifying its sources is an important step for improving animal wellbeing, research outcomes, and study costs^15^.

The terminology in ethology and aggression theory is complex but precise, and we first begin with a brief overview (see examples at www.mousebehavior.org, and^15,19^). Agonistic interactions occur between two or more animals in order for one animal to gain control of a future or currently available resource^20^. These interactions can be further classified by context (territorial versus dominance behaviors), or by outcome (mediated *versus* escalated aggression). Fighting is always risky—a losing animal may be injured, castrated, or die—and so animals often engage in ritualized conflicts and détente agreements to avoid these risks. Territories can be thought of as agreements in space, where territory holders have absolute control over resources within the space. Territorial behaviors do not require social relationships, and can be communicated via simple cues. In mice, territory boundaries are marked with odor cues, resident territory holders threaten intruders with “tail rattle” and “zig-zag” behaviors, and intruders submit by fleeing the territory boundaries^21,22^. Conversely, dominance hierarchies can be thought of as agreements in time, where dominant animals have priority access to resources. These behaviors require social relationships, where group members must recognize each other, and a much greater complexity in social cognition. For instance, dominance rank can shift with motivational changes, with differing resource availability, and between different kinds of resources. In mice, these relationships are again maintained with odor cues, dominant animals threaten subordinates with “mounting” and “sideways-threat” behaviors, and subordinates submit either by fleeing or with a characteristic submission posture^21,22^. When an intruder or subordinate successfully avoids fighting, and ends an agonistic reaction by fleeing or submitting, the interaction is referred to as “mediated aggression”. In mice, mediated aggression comprises the vast majority of agonistic interactions and is essential in preventing agonistic encounters from progressing to mortality^19^. However, an intruder or subordinate, may respond to a threat with a retaliatory bite, at which point both animals display a range of aggressive behaviors. This “escalated aggression” or “fighting” is potentially injurious, and ends with either a submission, a flee, or the ultimate incapacitation of one combatant. Conditions that make it possible for mediated aggression to be effective in limiting escalated aggression are the ultimate goals for good behavioral management.

Few mouse aggression studies have examined spontaneous home-cage aggression. Instead researchers typically induce territorial aggression by singly housing males then introducing an intruder. Since territorial and dominance aggression involve very different threat and submission behaviors in mice, these studies provide limited information^15,16^. Consequently, very little is known about genetic and environmental factors contributing to home-cage aggression, especially with regards to the escalation into fighting (for review see:^15^). Furthermore, the controlled scientific literature on laboratory mouse home-cage aggression, is spotty and inconsistent, with few experimental results replicating. One explanation for this is that home-cage aggression is so multifactorial that each controlled study only sees one tiny piece of the puzzle and other multifactorial effects either hide or exaggerate the effect of the factor under study^15^. This is exactly the situation where an epidemiological study is much more powerful and provides much stronger inference than a controlled study^23^. Therefore, our group conducted a year-long epidemiologic study identifying potential factors influencing fighting in male mice in a variety of research protocols and housing environments. This study aimed to 1) quantify the point-prevalence of fighting (escalated aggression) and related trauma across our institution; and 2) identify potential risk factors associated with fighting.

Behavioral epidemiology provides a means to test the relevance of previous findings in a real-world setting, to test novel hypotheses, and to control for multifactorial effects. A particular strength of the approach is that epidemiological studies are “statistically controlled”, which provides the ability to identify the effect of factors in a sea of multifactorial influences—i.e. all risk factors are tested in a single analysis, and each is tested after controlling for the noise introduced by all other confounding and risk factors^23,24^). The weakness of the approach is that only risk factors that vary and are measured can be tested, and false positive results can arise when too many risk factors are measured. Thus, this approach is most powerful when hypothesis-driven^23^. Accordingly, we first identified risk factors previously related to fighting in laboratory mice, and additional risk factors that had clear a priori hypotheses. Given the paucity of replicated results for home-cage aggression^15^, we included factors that have been experimentally associated with stress and/or abnormal behavior, as abnormal behavior is often an indicator of chronic stress, and stress and aggression are inextricably linked^25,26^.

It is received wisdom in mouse husbandry that there are strong differences between mice strains in home-cage fighting. However, the formal literature supporting this is sparse. Thus, aside from a lack of difference between C57BL/6 substrains^16^, and a study reported after ours was completed^27^, we are unaware of any formal comparison of home-cage fighting between mouse strains. In contrast strain, sex, genotype, hormonal, physiological, and behavioral state effects on territorial aggression in resident/intruder tests are well documented (e.g.^28–30^).

At the cage level, multiple studies suggest higher stocking densities increase aggression and fighting^31–34^, although recent work in modern caging systems complicates this story^35^. This effect seems to be driven not by cage size or density per se, but by the absolute number of mice in the cage^15,31^. There is less work on the effects of housing systems on fighting in laboratory mice, despite largescale adoption of individually ventilated cages (IVC) and corncob bedding. In fact, both are potential risk factors for fighting. For instance, in choice tests BALB/c mice avoid individually ventilated cages (IVC) and prefer static cages^36^. Corncob bedding contains biologically active estrogen disruptors, which suppress estrogen receptor expression, and induce territorial aggression in deer mice (*Peromyscus*)^37^. Environmental enrichment shows variable results. For example, huts or other cage furniture can either increase, decrease, or have no effects on fighting^38–40^. Provision of appropriate nesting material in suitable amounts affects thermal stress and behavior^40^ with potential effects on fighting. Furthermore, transferring nesting material during cage-change can influence fighting^41^. Finally, animals in different cages may undergo very different procedures, and potentially painful identification methods can increase fighting^16^, leading to the idea that the same may be generally true of any potentially painful procedure^15,42^.

Macroenvironmental factors can have significant consequences on laboratory mouse behavior. Consistent with observations in wild mice^43^, higher ambient temperature increases territorial aggression in two inbred strains of mice^30^. Seasonal effects on aggression can be seen in multiple laboratory mammalian species^44–47^, but seasonality of aggression has not been identified in laboratory mice (although seasonality of abnormal behavior is seen^48^). Mice housed higher on the rack show evidence of increased stress in terms of stress physiology, immune function, and abnormal behavior^49,50^. Similar effects in mice and other species can manifest in terms of room position with respect to human traffic^51,52^.

Many aspects of laboratory mouse biology and husbandry have been experimentally or anecdotally associated with changes in aggression, abnormal behavior, and stress. Unfortunately, the picture of how these factors influence levels of home-cage aggression and fighting is incomplete. An epidemiological study allows a holistic approach to assessing potential factors and promotes hypothesis formation for further study. We predict that the factors reviewed above (strain, higher numbers of mice per cage, IVC caging, corncob bedding, lack of nesting enrichment, presence of shelter enrichment, ear notches or punches, potentially painful procedures or morbidities, higher room temperature, season, higher cage row on the rack, and cages exposed to human traffic) will be associated with a greater prevalence of fighting.

## Results

### Population data and prevalence

In total, 2679 cages were observed in 43 rooms distributed between 9 different housing facilities. Of those cages, 1105 were male-only cages, 983 were female-only, and 591 were mixed male and female. Of the 1105 male-only cages observed, 841 contained multiple males housed together. Only a single event of fighting was observed in an all-female cage and similarly in a mixed male and female cage. Fighting behaviors were observed in 116/841 (13.8%) of multi-male cages, 16/116 (13.8%) of which had signs of trauma visible cage-side. Every cage containing animals with fight wounds showed fighting behaviors.

### Risk factors for fighting

**Figure 1:**
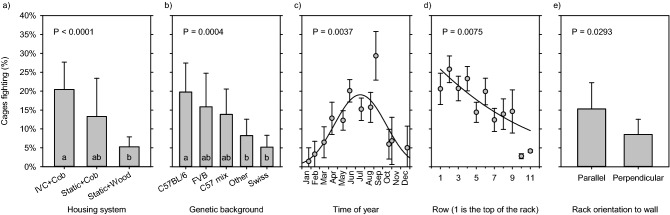
Risk factors that significantly predict fighting. (**a**) Cage type and bedding. (**b**) Strain. (**c**) Time of year. (**d**) Cage row. (**e**) Rack orientation. The P-value of each risk factor is shown. For categorical outcomes LSM (i.e. the mean controlling for all other effects in the model) and SE are calculated and inverse-link transformed to real world values. Tukey post-hoc tests are shown by letters within each bar, where bars with the same letter do not differ significantly. For continuous effects, an equivalent LSL (least-squares line) is calculated at the mean value of all other effects in the model; then for each data point a residual is calculated and added to the LSL. These corrected observed values are then averaged and the resulting mean and SE are plotted.

Risk factors are presented in order of influence on the model (LogWorth). Housing system significantly predicted fighting (Likelihood-Ratio χ^2^ = 23.07; DF = 2; P < 0.0001; Fig. 1a), with mice in IVC cages with corncob bedding showing significantly higher levels of fighting than those housed in static cages on wood chips (Tukey adjusted P < 0.0001). Background strain significantly predicted fighting (LR χ^2^ = 20.67; DF = 4; P = 0.0004; Fig. 1b). Time of year significantly predicted fighting with a cyclical pattern (Quadratic combined LR χ^2^ = 11.18; DF = 2; P = 0.0037; Fig. 1c), with fighting being most likely during summer, and lowest during winter months. Cage row significantly predicted fighting (LR χ^2^ = 7.144; DF = 1; P = 0.0075; Fig. 1d), with fighting decreasing in a linear fashion from the top of the rack to the bottom of the rack. Finally rack orientation significantly predicted fighting (LR χ^2^ = 4.752; DF = 1; P = 0.0293; Fig. 1e), with fighting being more likely in racks oriented parallel to the wall of the room than racks oriented perpendicular to the wall.

**Figure 2:**
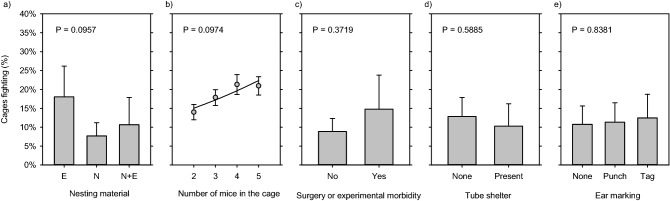
Risk factors that do not significantly predict fighting. (**a**) Nesting material (E = Envirodri; N = Nestlets). (**b**) Number of mice in the cage. (**c**) Surgery or experimental morbidity. (**d**) Tube shelters. (**e**) Ear marking. Data are summarized and plotted as described for Fig. 1.

As shown in Fig. 2, the remaining factors all failed to significantly predict fighting. These were: type of nesting material (LR χ^2^ = 4.692; DF = 2; P = 0.0957; Fig. 2a); number of mice in the cage (LR χ^2^ = 4.692; DF = 1; P = 0.0974; Fig. 2b); surgery or experimentally-induced morbidity (LR χ^2^ = 0.7973; DF = 1; P = 0.3719; Fig. 2c); shelter enrichments (LR χ^2^ = 0.2927; DF = 1; P = 0.5885; Fig. 2d); and ear marking (LR χ^2^ = 4.692; DF = 2; P = 0.3533; Fig. 2e).

## Discussion

To our knowledge this is the first 12-month epidemiological study of the prevalence of fighting in mice. Fighting was almost exclusively confined to male mice, occurring in 14% of cages. Trauma was visible in only 14% of cages in which fighting behavior was actually observed. Accordingly, 1.9% of cages observed showed trauma visible from cage-side checks. This compares well with recent multi-site survey data from Europe, which reported a prevalence of 2.9% of cages with trauma visible from cage-side checks^27^. In the 86% of cages where fighting was observed but no trauma was noted, fighting may have not yet resulted in related injuries or the observed fighting may have been an acute rather than ongoing event. More likely, however, is that wounding is not easily appreciated by visual examination from outside of the cage. For instance, subcutaneous scoring of pelts is a more sensitive predictor of bite trauma than exterior physical exams^18^. Regardless, a critical take-home message from this study is that the industry standard of using trauma visible from the outside of the cage misses many of the cages fighting is actually occurring. Furthermore, given that mice were observed during the day (when they would normally be asleep), and most fighting occurs at night^19^, our behavioral observations most likely underestimated the proportion of mice fighting. Indeed, the fact that mice would fight when they would normally be asleep only underscores the severity of the problem.

In the current study the single greatest predictor of fighting or injury was housing system. Individually ventilated cages bedded with corncob had significantly higher levels of fighting than static caging bedded with woodchips. However, as we found no IVC cages bedded with woodchips, and only a small number of static cages bedded with corncob, we could not separate the effect of these two variables. Answering this question is the focus of an ongoing follow-up controlled study.

Studies of the impact of IVC housing on mouse wellbeing have generally focused on anxiety, stress, and mouse preference, rather than fighting, and IVC housing clearly has several behavioral effects on mice. Anxiety-related behaviors (including reduced activity levels) are increased in C3H and C57BL/6 mice housed in IVCs when compared to traditional static caging^53^. Mice prefer cages with lower (or no) ventilation rates^36^ and high ventilation rates induce cold stress^54^. Sound intensity levels at multiple frequencies are significantly higher in ventilated cages when compared to static cages^55^, although these may not be audible to mice^56^. IVC racking also vibrates at frequencies perceptible to mice^57^. Unsurprisingly, breeding performance is affected as a result, with IVC systems showing greater variability^58^. Additionally, strain differences in behavioral phenotyping measures in mice housed in static caging are not replicable in IVC caging, and vice-versa^59^. In line with this evidence that IVCs are stressful to mice, recent multi-site survey data also identifies IVC housing as a risk factor for aggression in mice^27^.

Similarly, bedding affects the behavior and wellbeing of mice. Mice show a strong preference for wood-based bedding materials and aversion to corncob when given a choice between bedding materials^60^. Furthermore, corncob bedding contains multiple estrogen-disrupting compounds, namely tetrahydrofurandiols (THF-diols) and leukotoxin-diols (LTX-diols)^61–63^. These diols disrupt cyclicity and breeding behavior in female rats and reduce mounting behavior in male rats^63,64^. Resident male California mice (*Peromyscus californicus*) are more aggressive to intruders when housed on corncob bedding, and females raised on corncob bedding are less likely to exhibit social withdrawal after social defeat^37,65^. Considering that corncob appears to affect aggressive and affiliative behaviors in rodents, its appropriateness as direct-contact bedding for laboratory mice should be examined.

After housing system, genetic background was the next strongest predictor of fighting. Historically, SJL, DBA, FVB, and BALB/c have all been identified as strains with high levels of inter-male aggression^14,28,31,66^. However, most studies of strain differences in aggression employ pairwise or three-way comparisons, making it difficult to obtain a complete picture of which strains are the most and least aggressive, especially as this work typically examines territorial aggression. To our knowledge such studies have not placed C57BL/6 mice as one of the most aggressive strains. This is consistent with our (JPG, BNG, KPC) experience breeding and maintaining C57BL/6 mice in a variety of facilities all of which used static caging and woodchip bedding, and where fighting was rarely if ever seen in C57BL/6 mice. Similarly, we were unable to find any differences between the C57BL/6 substrains when housed in ventilated caging and woodchip bedding in follow-up studies^16^. Further consistent with this interpretation, recent data from the UK, where corncob is rarely used, finds C57BL/6 mice to be amongst the least aggressive strains^27^. Finally given the overlap between strains (in terms of Tukey tests), and that fact that C57BL/6 crosses do not differ from any strain, it would be incorrect to state that C57BL/6 mice are more aggressive than other strains in general.

The prevalence of fighting varied by almost 20 percentage points with time of year. The prevalence of ulcerative dermatitis (self-injurious scratching behavior in mice^11,67,68^) also varies with time of year^48^. While lab mouse behavior changes with time of year, whether this reflects a biologically normal response to time of year, or a response to other environmental cues is unclear. Mice may have a year-based internal clock associated with seasonal activities in the wild, such as dispersion in the spring^69^. Given their magnetic sense^70^, they may be sensitive to fluctuations in the Earth’s magnetic field associated with season, or may be exposed to seasonal environmental factors via caretakers. The relatively cyclical effect of time of year supports this interpretation. Mice may also be responding to seasonal influences on human behavior. For instance, in our data, gathered at an academic institution, we see a spike in aggression in September, which corresponds to the start of the school year in the United States, and a seasonal peak influx of new investigators and attendees of our new-investigator mouse handling workshop.

The final two predictors of fighting, cage location on the rack and orientation of rack in the room, are both examples of how small variations in housing location can affect behavior and physiology in experimentally significant ways. Mice housed on higher rows in the rack show evidence of increased stress, including changes in stress physiology, immune function, and abnormal behavior^49,51^. Animals on higher rows may be exposed to greater noise and vibration in IVC housing systems (due to proximity to the fans and blowers necessary for ventilation), and to brighter lights. For instance, light levels can be 24 times greater at the top of a rack compared to the bottom^71^. Similarly, racks oriented parallel to the wall may be more exposed to bright light. Cages on these racks are all exposed to human traffic, while on racks oriented perpendicular to the wall, only the outmost cages are exposed to similar levels of human traffic. As discussed above, exposure to human traffic is related to abnormal behavior in other species^39,51,52^.

Multiple factors tested in this model did not appear to be significant predictors of fighting despite past research suggesting otherwise. Of particular note was the lack of a significant effect of the number of mice in the cage, which previous studies have shown to be the key factor underlying increased aggression at higher stocking density^31,32^, but see^35^ for a counter-example. Our failure to observe this effect may be related to our SOP for injured mice. When fight wounds are observed by the care staff at our institution, these mice are separated into smaller groups. This usually occurred early in the day during animal health checks by staff and before our observations were performed. This would decrease the prevalence of higher-density cages and observations of aggression at higher densities and artificially inflate aggression at lower stocking densities. Similar confounds are proposed in a recent survey study^27^.

We did not observe any effects of environmental enrichment (tubes) in the current study. All mice are provided with nesting material in our facility, so we could not assess the impact of the absence of nesting material. Similarly, nesting material is transferred at cage change as part of our routine husbandry and could not be tested. The type of nesting material did not have a significant impact. While nesting material is typically found to be beneficial for mouse wellbeing^5^, the evidence for shelters and cage furniture is mixed^15,38–40,72^, with severe increases in aggression reported in some cases^19^. Fine-grained cues and behavioral opportunities (such as the ability to successfully flee an aggressor), most likely are driving these differences, but they are poorly understood at this time^15^. Accordingly, while cardboard tubes did not increase aggression in this study, we still caution their use without close monitoring for increases in fighting.

Given the role of pain in promoting aggression in many species, including mice and rats^42^, we have speculated that husbandry or experimentally induced pain might make mice more aggressive^15^. Identification methods are well documented to affect physiology and behavior, and ear notching did increase aggression in more than one study^16,27^. Neither the presence of ear tags, nor research-related morbidities significantly predicted fighting in mice. The transient nature of the discomfort caused by placing an ear tag and the typical time at which tags are placed, at weaning, may preclude observations of aggression in our data. In terms of pain associated with other surgical manipulations and tumor induction, adequate analgesia or, conversely, sickness behavior may be the reason for reduced signs of aggression. It might also be that animals were observed during daytime, and thus not observed during their active period when they might be most influenced by potential pain.

As we hypothesized, fighting in laboratory mice is multifactorial and an epidemiological approach provides insight into potential factors. However, epidemiological studies are always somewhat constrained by the population studied. For instance, the absence of ventilated caging with wood shavings in our data made it impossible to separate the effects of bedding from caging system. Teasing apart confounding variables such as these is possible with controlled studies, now underway at our institution. It is sobering that of the 5 factors affecting fighting (housing system, background strain, time of year, cage row, and rack orientation), only corncob bedding is easily addressed. The remaining factors are difficult-to-impossible to remediate in a research institution due to research, economic, temporal, and spatial constraints. Furthermore, housing system (IVCs and corncob) and rack orientation are often arbitrary changes imposed on facilities by engineers and architects, without any evidence-based consideration of the welfare of mice. If we are serious as an industry about long-term improvement to mouse wellbeing, then housing and husbandry decisions need to be driven by evidence gathered by experts in mouse wellbeing (including behavior), and not be decisions driven by expediency or cost alone.

## Conclusion

When examining potential predictors of fighting in laboratory mice, many factors that were reported in the experimental literature were not replicated. Strains anecdotally reported as non-aggressive, such as C57BL/6 mice, were found to be more likely to fight than Swiss lineage mice, which includes the historically aggressive SJL strains^66,73^. Also, cardboard tubes and discomfort were not predictors of fighting despite some studies reporting the opposite. Many of the factors that do influence fighting, like strain, season, and housing location, can be taken into account when designing experimental manipulations but cannot be controlled. For example, males of C57BL/6 background may be less likely to fight on a bottom row during the winter months, but strict prescriptions as to genetic background and housing would be unnecessarily onerous. However, the single greatest predictor of fighting, housing system, is a factor that could conceivably be addressed to minimize aggression, though this may only be practical when a facility is renovated or newly built. Often, housing systems are chosen for cost effectiveness, human perception of greater cleanliness, and ease of handling, while animal welfare is overlooked. This study emphasizes the importance of assessing the welfare costs of housing system choices, and that fighting-related morbidities and research confounds may nullify any of the perceived positive aspects these housing systems may have in terms of operational or financial expediency.

## Materials and methods

### Ethical oversight

This was an epidemiological study of mice housed at Stanford University, an AAALAC accredited animal care and use program. This study was performed under an animal care and use protocol approved by Stanford’s Institutional Animal Care and Use Committee (IACUC). In addition, since this was an observational study, every cage of mice was also assigned to one of multiple distinct protocols also approved by the university’s IACUC, spanning many areas of biomedical research (e.g. cardiology, neurology, toxicology, oncology, genetics). All methods were carried out in accordance with relevant guidelines and regulations.

### Housing and husbandry

All mice were housed in one of nine vivaria on the Stanford University main campus. Every solid-bottomed, contact-bedded cage housed one to five adult mice. Male and female mice were generally housed separately unless breeding. Health status varied by room and included SPF barrier facilities, conventional housing, and biohazardous facilities. Mice were either bred in-house or came from various vendors, including internationally. All animals housed within the barrier facility were bred or re-derived within the facility.

In general, all mice were housed in 12:12 light:dark light cycles at room temperatures ranging between 20 °C and 26 °C and humidities between 30 and 70%. Mice housed on individually ventilated caging systems were housed in either Innovive Disposable IVC Rodent Caging Systems (523 cm^2^ floorspace, Innovive, San Diego, CA) or Max 75 Plastic Mouse Cages (537 cm^2^ floorspace, Alternative Design, Siloam Springs, AR). Static housed mice were housed in either Allentown Plastic Caging (563 cm^2^ floorspace, Allentown, Inc., Allentown, NJ) or Innovive disposable IVC caging with static filter tops. Cage changes occurred once every two weeks for IVC caging and once a week for static caging. All cages, independent of cage type, were stored on racks that were either parallel or perpendicular orientation to a wall of a rectangular shaped room. Racks ranged in size from 5 to 11 rows and 7 to 8 columns. Manufacturers of racks were the same as the cages they held with the exception of static Innovive caging, which were housed on Allentown static racks. Bedding included 7092–7097 Teklad corncob bedding or 7090 Teklad sani-chips (Envigo, Madison, WI). Nesting material included either approximately 6–8 g of Envirodri (Shepherd Specialty Papers, Watertown, TN), 1–2 Nestlets (Ancare, Bellmore, NY), or both. Nests were generally transferred to clean cages during cage change. Cardboard tubes (Custom Paper Tubes, Cleveland, OH) were also provided to some mice as needed, subjectively determined by research, veterinary, or care staff. Mice were fed ad libitum 2018 Teklad 18% protein rodent diet (Envigo, Madison WI) and provided ad libitum water. Mice housed in Innovive caging received Aquavive Mouse Pre-filled Acidified Water Bottles (Innovive, San Diego, CA). All other mice received reverse osmosis treated water filled on site and provided in either Allentown or Alternative Design water bottles. Exceptions to normal feed and water were protocol specific and included a variety of custom diet formulations and water solutions that were either made in-house or purchased from specialty vendors.

### Epidemiological design

The cage was the sampling unit and the experimental unit. This study was a cross-sectional study designed to assess point-prevalence of fighting as a function of time of year with each cage observed only once. Such a design cannot give incidence rates, but is protected against the complications inherent to longitudinal designs (such as subject attrition). We first performed a one-month pilot study, which allowed for troubleshooting and refinement of sampling techniques, identification of any overlooked factors, and feasibility assessment. These pilot data were not analyzed further. Data collection for this study began in April of 2017 and continued through March of 2018.

Formal a-priori power calculations do not exist for the advanced analytical methods employed here, and alternative approaches for similar methods with continuous outcomes (specifically Mead’s Resource Equation)^74^ do not apply to categorical outcomes. Instead, we based our target sample size (of approximately 3000 cages) on previous epidemiological studies in mice^50,75^.

### Sampling, and inclusion and exclusion criteria

Initially, all rooms and cage racks on central campus were considered for inclusion in this study, including barrier facilities. Then all rooms kept under a non-standard light cycle were excluded so that observations were not performed in the dark. Because epidemiological studies can only test for the effects of factors that vary, facilities and rooms to be sampled were chosen to maximize the variability of the variables of interest. Additional care was taken to ensure that other factors of interest (e.g. rack orientation), or nuisance variables (e.g. facility) were balanced across time of year. After this filtering, the rooms available for sampling on any given day were chosen at random. Racks and rack sides were chosen at random within rooms taking care that any given rack side was sampled only once over the course of the entire study.

All data were collected by the same investigator (JHT). After a rack side was selected, the starting row was randomly selected using a spinning wheel, modified dice, or coin flip to avoid confounding rack row with order of observation. Sampling began at the rack column closest to the wall and continued horizontally across the row. When a row was completed, the row directly below was then sampled in the same fashion. After reaching the bottom row, sampling resumed on the top row and worked its way down until the starting row had been reached. For racks that were parallel to the wall, sampling began at the left hand column. Sampling was performed by gently removing cages one by one and assessing animals and environmental factors visible from outside the cage. Mice were not handled. Information from the cage cards was also collected when available.

To avoid any possible bias, or missing important unexpected information (for instance prior to the epidemiological data we collected on barbering, self-barbering was unknown, and barbering was assumed to be directed to cagemates only^75^), it is important to avoid assumptions and collect data in an unbiased manner. Therefore data were collected from all cages on the rack side chosen. Singly housed cages were recorded but excluded from analysis. Due to the lack of fighting in female and breeding cages, data were recorded for these cages but excluded from further analysis.

### Primary outcome

The primary outcome variable for this study was fighting. Fighting was assessed in two ways. First trauma consistent with escalated aggression (“fight wounds”) was noted during the visual inspection of each cage. Second, as it is unclear whether fight wounds reliably identify the majority of cages which are actually engaged in fighting behavior, after completing visual inspection of the cages, the rack side was visually monitored for an additional 5 min by scanning all cages repeatedly from top to bottom and side to side. For these observations “behavioral fighting” was defined as an aggressive event where one mouse exhibited threat or attack behaviors such as mounting or biting another mouse, and the second mouse exhibited retaliatory biting such that the aggressive event escalated into bilateral physical aggression. Such escalated aggression can ultimately lead to injury^19^. These cases were first noted via auditory (vocalization, substrate dispersion) and visual cues (turbulent water bottles, increased activity levels) throughout the sampling process. For a cage to fit a conservative criterion of fighting, these escalated aggression interactions had to be observed on two different occasions for a period of greater than one second.

### Independent variables

Date of sampling was recorded for every cage. At the room level, facility name, room number, floor area, high and low temperatures over the last 24 h, and high and low humidity over the last 24 h were recorded.

At the rack level, the rack number, rack side, the position of the rack (parallel to wall vs. perpendicular to wall, parallel to room entrance versus perpendicular to entrance), distance rank to the entrance (e.g. 4th rack from the door), direct visual exposure to the entryway, and days since the last cage change were recorded.

At the cage level, all data was collected either through visual inspection from outside the cage or through information on cage cards, with the exception of background strain. Background strain was recorded using information from the cage cards, protocol information, or direct communication with the research group using the mice. Location on the rack (column and row number), housing type, bedding type, nesting material provided, whether cage furniture was present or not, dietary changes, the number of animals in the cage (not including pups), the sex of the adult animals in the cage (male, female, or both), the presence of pups (yes or no), the presence of ear markings (ear punches or ear tags), and any other morbidities found in the cage were recorded for every cage sampled.

### Data processing and statistical analysis

Prior to data analysis we excluded cages from the data set where aggression could not (singly housed mice) or did not occur (female mice and breeding mice). (See results for details). Data were initially analyzed as a logistic regression implemented as a Generalized Linear Model in JMP PRO 14. Significance was tested with likelihood ratios, following best practice for logistic regression^76^. The advantage of this approach is that all hypothesized risk factors are tested in a single analysis, and the effect of all other risk and confounding factors are controlled for when testing each risk factor. This allows the detection of subtle effects that would otherwise be drowned out by predictable noise introduced by other risk factors. It also allows for the detection of collinearity (excluding risk factors that are superficially significant only because they happen to be correlated with a more important risk factor).

In order to minimize the risk of false-discovery, we limited ourselves to predicted hypothesis-driven risk factors for fighting. We then identified the variables which best captured each hypothesis while minimizing collinearity with other variables. When variables were collinear we combined or simplified them if possible, or excluded them if necessary to maintain model integrity. When we had to exclude variables we chose the variable that disrupted the model without attaining significance, or the variable least likely to have explanatory power based on its lack of variance, or the variable with least influence and/or greatest collinearity as indicated by LogWorth. This follows best practice for logistic regression model design^76^ and epidemiological analysis. Accordingly, strain was simplified to background strain family. Cage type and bedding type were combined into a single variable (housing system) because there were no IVC cages with woodchip bedding. Temperature and humidity disrupted the model, and were excluded in favor of keeping time of year and facility in the model (i.e. temperature and humidity might well contribute to the time of year effect, but time of year had additional explanatory power, and was therefore retained over retaining temperature and humidity). Variables describing room layout and human traffic tended to occur in very specific combinations which were room- or facility-specific, so we used only rack orientation in each room. The presence of surgical intervention, experimentally induced morbidities or other potentially painful manipulations were combined into a simple yes/no category. Other variables: day of year, row on rack, ear marking, number of mice, nesting material type, and presence/absence of shelter enrichment were included as-is. Day of year was modelled as a quadratic function, and tested with a custom joint test of the linear and quadratic terms. Including non-hypothesized interactions is highly unadvisable in logistic regression as spurious false positive results are very likely, especially when the case:control ratio deviates from 50% (as it does here). Our only planned interaction to test was Cage type by Bedding type. Given that this was not possible, and we had no other hypothesized interactions, no further interactions were included in the model. We ran the model including facility as a stratifying variable which formally tests for within-facility effects, and excluding facility which formally tests for between-facility effects. The two models yielded the same results, and facility was confounded with many variables and induced quasi-complete separation. Therefore following best practice for logistic regression^76^ we present the model excluding facility.

While JMP is ideal for data visualization and model design, SAS provides more powerful options for post-hoc testing. Thus, to estimate mean effects, and perform post-hoc tests, the same analyses were then performed using PROC GENMOD in SAS 9.3 for Windows. Post-hoc tests were Tukey-corrected as appropriate to control for multiple testing.

## References

[1] Würbel H, Garner JP (2007). Refinement of rodent research though environmental enrichment and systematic randomization. NC3Rs.

[2] Gaskill BN (2012). Heat or insulation: behavioral titration of mouse preference for warmth or access to a nest. PLoS ONE.

[3] Gaskill BN (2013). Impact of nesting material on mouse body temperature and physiology. Physiol. Behav..

[4] Gaskill BN, Karas AZ, Garner JP, Pritchett-Corning KR (2013). Nest building as an indicator of health and welfare in laboratory mice. J. Vis. Exp..

[5] Gaskill BN (2013). Energy reallocation to breeding performance through improved nest building in laboratory mice. PLoS ONE.

[6] Gaskill BN, Rohr SA, Pajor EA, Lucas JR, Garner JP (2011). Working with what you’ve got: changes in thermal preference and behavior in mice with or without nesting material. J. Therm. Biol.

[7] Gaskill BN, Winnicker C, Garner JP, Pritchett-Corning KR (2013). The naked truth: breeding performance in nude mice with and without nesting material. Appl. Anim. Behav. Sci..

[8] Hess SE (2008). Home improvement: C57BL/6 mice given more naturalistic nesting materials build better nests. J. Am. Assoc. Lab. Anim. Sci..

[9] Jirkof P (2014). Burrowing and nest building behavior as indicators of well-being in mice. J. Neurosci. Methods.

[10] Rock ML (2014). The time-to-integrate-to-nest test as an indicator of wellbeing in laboratory mice. J. Am. Assoc. Lab. Anim. Sci..

[11] Adams SC, Garner JP, Felt SA, Geronimo JT, Chu DK (2016). A "Pedi" cures all: toenail trimming and the treatment of ulcerative dermatitis in mice. PLoS ONE.

[12] Marx JO, Brice AK, Boston RC, Smith AL (2013). Incidence rates of spontaneous disease in laboratory mice used at a large biomedical research institution. J. Am. Assoc. Lab. Anim. Sci..

[13] Deacon RM (2006). Housing, husbandry and handling of rodents for behavioral experiments. Nat. Protoc..

[14] Whary MT, Baumgarth N, Fox JG, Barthold SW, Fox JG (2015). Ch. 3. Laboratory Animal Medicine.

[15] Weber EM, Dallaire JA, Gaskill BN, Pritchett-Corning KR, Garner JP (2017). Aggression in group-housed laboratory mice: why can't we solve the problem?. Lab. Anim. (NY).

[16] Gaskill BN (2017). The effect of early life experience, environment, and genetic factors on spontaneous home-cage aggression-related wounding in male C57BL/6 mice. Lab. Anim. (NY).

[17] Blankenberger WB (2018). Breaking up is hard to do: does splitting cages of mice reduce aggression?. Appl. Anim. Behav. Sci..

[18] Gaskill BN (2016). He's getting under my skin! Comparing the sensitivity and specificity of dermal vs subcuticular lesions as a measure of aggression in mice. Appl. Anim. Behav. Sci..

[19] Howerton CL, Garner JP, Mench JA (2008). Effects of a running wheel-igloo enrichment on aggression, hierarchy linearity, and stereotypy in group-housed male CD-1 (ICR) mice. Appl. Anim. Behav. Sci..

[20] Scott JP, Fredericson E (1951). The causes of fighting in mice and rats. Physiol. Zool..

[21] McAllister KH, Dixon AK (1989). Reappraisal of the mouse ethogram according to grant and mackintosh—social and aggressive-behavior. Aggressive Behav..

[22] Grant EC, Mackintosh JH (1963). A comparison of the social postures of some common laboratory rodents. Behaviour.

[23] Woodward M (1999). Epidemiology: Study Design and Data Analysis.

[24] Grafen A, Hails R (2002). Modern Statistics for the Life Sciences.

[25] Koolhaas JM (1999). Coping styles in animals: current status in behavior and stress-physiology. Neurosci. Biobehav. Rev..

[26] Veenema AH, Meijer OC, de Kloet ER, Koolhaas JM, Bohus BG (2003). Differences in basal and stress-induced HPA regulation of wild house mice selected for high and low aggression. Horm. Behav..

[27] Lidster K, Owen K, Browne WJ, Prescott MJ (2019). Cage aggression in group-housed laboratory male mice: an international data crowdsourcing project. Sci. Rep..

[28] Canastar A, Maxson SC (2003). Sexual aggression in mice: effects of male strain and of female estrous state. Behav. Genet..

[29] Nelson RJ, Chiavegatto S (2000). Aggression in knockout mice. ILAR J..

[30] Greenberg G (1972). The effects of ambient temperature and population density on aggression in two inbred strains of mice, *Mus musculus*. Behaviour.

[31] Van Loo PL, Mol JA, Koolhaas JM, Van Zutphen BF, Baumans V (2001). Modulation of aggression in male mice: influence of group size and cage size. Physiol. Behav..

[32] Bailoo JD (2018). Evaluation of the effects of space allowance on measures of animal welfare in laboratory mice. Sci. Rep..

[33] Smith AL, Mabus SL, Muir C, Woo Y (2005). Effects of housing density and cage floor space on three strains of young adult inbred mice. Comp. Med..

[34] Poole TB, Morgan HD (1973). Differences in aggressive behaviour between male mice (*Mus musculus* L.) in colonies of different sizes. Anim. Behav..

[35] Jirkof P (2020). The effect of group size, age and handling frequency on inter-male aggression in CD 1 mice. Scientific reports.

[36] Baumans V, Schlingmann F, Vonck M, van Lith HA (2002). Individually ventilated cages: beneficial for mice and men?. Contemp Top Lab Anim Sci.

[37] Villalon Landeros R (2012). Corncob bedding alters the effects of estrogens on aggressive behavior and reduces estrogen receptor-alpha expression in the brain. Endocrinology.

[38] Marashi V, Barnekow A, Ossendorf E, Sachser N (2003). Effects of different forms of environmental enrichment on behavioral, endocrinological, and immunological parameters in male mice. Horm. Behav..

[39] Ambrose N, Morton DB (2000). The Use of Cage Enrichment to Reduce Male Mouse Aggression. J. Appl. Anim. Welfare Sci..

[40] Lockworth CR, Kim SJ, Liu J, Palla SL, Craig SL (2015). Effect of enrichment devices on aggression in manipulated nude mice. J. Am. Assoc. Lab. Anim. Sci..

[41] Van Loo PLP, Kruitwagen CLJJ, Van Zutphen LFM, Koolhaas JM, Baumans V (2000). Modulation of aggression in male mice: Influence of cage cleaning regime and scent marks. Anim. Welf..

[42] Ulrich R (1966). Pain as a cause of aggression. Am. Zool..

[43] Crowcroft, P. *Mice all over*. (Foulis, 1966).

[44] Theil JH, Beisner BA, Hill AE, McCowan B (2017). Effects of human management events on conspecific aggression in captive rhesus macaques (*Macaca mulatta*). J. Am. Assoc. Lab. Anim. Sci..

[45] Vandenbergh JG, Vessey S (1968). Seasonal breeding of free-ranging rhesus monkeys and related ecological factors. J. Reprod. Fertil..

[46] Bailey AM, Rendon NM, O'Malley KJ, Demas GE (2016). Food as a supplementary cue triggers seasonal changes in aggression, but not reproduction, *Siberian hamsters*. Physiol. Behav..

[47] Kastner D, Apfelbach R (1987). Effects of cyproterone acetate on mating behavior, testicular morphology, testosterone level, and body temperature in male ferrets in comparison with normal and castrated males. Horm. Res..

[48] Kastenmayer RJ, Fain MA, Perdue KA (2006). A retrospective study of idiopathic ulcerative dermatitis in mice with a C57BL/6 background. J. Am. Assoc. Lab. Anim. Sci..

[49] Ader DN, Johnson SB, Huang SW, Riley WJ (1991). Group-size, cage shelf level, and emotionality in nonobese diabetic mice—impact on onset and incidence of IDDM. Psychosom. Med..

[50] Garner JP, Dufour B, Gregg LE, Weisker SM, Mench JA (2004). Social and husbandry factors affecting the prevalence and severity of barbering (‘whisker trimming’) by laboratory mice. Appl. Anim. Behav. Sci..

[51] Garner JP, Meehan CL, Famula TR, Mench JA (2006). Genetic, environmental, and neighbor effects on the severity of stereotypies and feather picking in Orange-winged Amazon parrots (*Amazona amazonica*): an epidemiological study. Appl. Anim. Behav. Sci..

[52] Mason GJ, Mendl M (1997). Do the stereotypies of pigs, chickens and mink reflect adaptive species differences in the control of foraging?. Appl. Anim. Behav. Sci..

[53] Kallnik M (2007). Impact of IVC housing on emotionality and fear learning in male C3HeB/FeJ and C57BL/6J mice. Mamm. Genome.

[54] David JM, Knowles S, Lamkin DM, Stout DB (2013). Individually ventilated cages impose cold stress on laboratory mice: a source of systemic experimental variability. J. Am. Assoc. Lab. Anim. Sci..

[55] Perkins SE, Lipman NS (1996). Evaluation of microenvironmental conditions and noise generation in three individually ventilated rodent caging systems and static isolator cages. Contemp. Top. Lab. Anim. Sci..

[56] Reynolds RP, Kinard WL, Degraff JJ, Leverage N, Norton JN (2010). Noise in a laboratory animal facility from the human and mouse perspectives. J. Am. Assoc. Lab. Anim. Sci..

[57] Norton JN, Kinard WL, Reynolds RP (2011). Comparative vibration levels perceived among species in a laboratory animal facility. J. Am. Assoc. Lab. Anim. Sci..

[58] Tsai PP, Oppermann D, Stelzer HD, Mahler M, Hackbarth H (2003). The effects of different rack systems on the breeding performance of DBA/2 mice. Lab. Anim..

[59] Mineur YS, Crusio WE (2009). Behavioral effects of ventilated micro-environment housing in three inbred mouse strains. Physiol. Behav..

[60] Mulder JB (1975). Bedding preferences of pregnant laboratory-reared mice. Behav. Res. Methods Instrum..

[61] Markaverich B (2002). A novel endocrine-disrupting agent in corn with mitogenic activity in human breast and prostatic cancer cells. Environ. Health Perspect..

[62] Markaverich BM (2002). Identification of an endocrine disrupting agent from corn with mitogenic activity. Biochem. Biophys. Res. Commun..

[63] Markaverich BM (2005). Leukotoxin diols from ground corncob bedding disrupt estrous cyclicity in rats and stimulate MCF-7 breast cancer cell proliferation. Environ. Health Perspect..

[64] Mani SK, Reyna AM, Alejandro MA, Crowley J, Markaverich BM (2005). Disruption of male sexual behavior in rats by tetrahydrofurandiols (THF-diols). Steroids.

[65] Trainor BC (2013). Sex differences in stress-induced social withdrawal: independence from adult gonadal hormones and inhibition of female phenotype by corncob bedding. Horm. Behav..

[66] Crispens CG (1973). Some characteristics of strain SJL-JDg mice. Lab. Anim. Sci..

[67] George NM (2015). Antioxidant therapies for ulcerative dermatitis: a potential model for skin picking disorder. PLoS ONE.

[68] Dufour BD (2010). Nutritional up-regulation of serotonin paradoxically induces compulsive behavior. Nutr. Neurosci..

[69] Rowe FP, Berry RJ (1981). Biology of the House Mouse: Symposia of the Zoological Society of London.

[70] Muheim R, Edgar NM, Sloan KA, Phillips JB (2006). Magnetic compass orientation in C57BL/6J mice. Learn. Behav..

[71] Greenman DL, Bryant P, Kodell RL, Sheldon W (1982). Influence of cage shelf level on retinal atrophy in mice. Lab. Anim. Sci..

[72] Olsson A, Dahlborn K (2002). Improving housing conditions for laboratory mice: a review of 'environmental enrichment'. Lab. Anim..

[73] Taketo M (1991). FVB/N: an inbred mouse strain preferable for transgenic analyses. Proc. Natl. Acad. Sci. U S A.

[74] Gaskill BN, Garner JP (2020). Power to the people: power, negative results and sample size. J. Am. Assoc. Lab. Anim. Sci..

[75] Garner JP, Weisker SM, Dufour B, Mench JA (2004). Barbering (fur and whisker trimming) by laboratory mice as a model of human trichotillomania and obsessive-compulsive spectrum disorders. Comp. Med..

[76] Allison PD, SAS Institute (1999). Logistic Regression Using the SAS System: Theory and Application.

